# Identifying Novel Cell Glycolysis-Related Gene Signature Predictive of Overall Survival in Gastric Cancer

**DOI:** 10.1155/2021/9656947

**Published:** 2021-03-12

**Authors:** Xin Zhao, Jiaxuan Zou, Ziwei Wang, Ge Li, Yi Lei

**Affiliations:** ^1^Department of Urology, The Affiliated Hospital of Southwest Medical University, Luzhou, Sichuan 646000, China; ^2^Sichuan Clinical Research Center for Nephropathy, Luzhou, Sichuan 646000, China; ^3^Fuzhou Medical College of Nanchang University, Fuzhou, Jiangxi Province 344100, China; ^4^College of Life Sciences, University of Chinese Academy of Sciences, Beijing 100049, China; ^5^Department of Endocrinology and Metabolism, The Affiliated Hospital of Southwest Medical University, Luzhou, Sichuan 646000, China; ^6^Cardiovascular and Metabolic Diseases Key Laboratory of Luzhou, Luzhou, Sichuan 646000, China

## Abstract

**Background:**

Gastric cancer (GC) is believed to be one of the most common digestive tract malignant tumors. The prognosis of GC remains poor due to its high malignancy, high incidence of metastasis and relapse, and lack of effective treatment. The constant progress in bioinformatics and molecular biology techniques has given rise to the discovery of biomarkers with clinical value to predict the GC patients' prognosis. However, the use of a single gene biomarker can hardly achieve the satisfactory specificity and sensitivity. Therefore, it is urgent to identify novel genetic markers to forecast the prognosis of patients with GC.

**Materials and Methods:**

In our research, data mining was applied to perform expression profile analysis of mRNAs in the 443 GC patients from The Cancer Genome Atlas (TCGA) cohort. Genes associated with the overall survival (OS) of GC were identified using univariate analysis. The prognostic predictive value of the risk factors was determined using the Kaplan-Meier survival analysis and multivariate analysis. The risk scoring system was built in TCGA dataset and validated in an independent Gene Expression Omnibus (GEO) dataset comprising 300 GC patients. Based on the median of the risk score, GC patients were grouped into high-risk and low-risk groups.

**Results:**

We identified four genes (*GMPPA*, *GPC3*, *NUP50*, and *VCAN*) that were significantly correlated with GC patients' OS. The high-risk group showed poor prognosis, indicating that the risk score was an effective predictor for the prognosis of GC patients.

**Conclusion:**

The signature consisting of four glycolysis-related genes could be used to forecast the GC patients' prognosis.

## 1. Introduction

Gastric cancer (GC) is one of the most common malignancies throughout the world. Although the incidence of GC has been declined in recent year, GC remains one of the most aggressive malignant tumors that severely threaten human health [1, 2]. According to the statistics data, there were 951,600 newly diagnosed cases of GC and 723,100 deaths related to GC in 2012 [3]. At present, most of the GC patients have already been at the progressive stage upon diagnosis or have even missed the best timing for surgical resection [4]. GC patients at the progressive stage usually have a low five-year overall survival (OS) due to recurrence and metastasis. Even patients with the same degree of progression may differ in prognosis and treatment efficacy [4, 5]. Therefore, early diagnosis and prognostic evaluation of GC are highly important. Efforts should be made to look for useful biomarkers to evaluate the prognosis of GC patients and to identify potential high-risk GC patients.

In recent years, a variety of biomarkers have been used as prognostic predictors of the GC patients. For example, the high expression of ANKRD49 is correlated to the size, infiltration, and metastasis of GC and facilitates the progression and poor prognosis of GC patients [6]. Besides, Tumor Necrosis Factor Receptor Superfamily Member 11B (TNFRSF11B) can significantly promote GC cell proliferation, migration, and invasion while inhibiting the apoptosis of the GC cells by activating the Wnt/*β*-catenin signaling pathway in GC cells. As a result, the survival of GC patients is lowered [7]. In addition, study has shown that JMJD2A regulates the growth of GC and high expression of JMJD2A predicts poor overall survival. Therefore, JMJD2A can serve as an independent prognostic factor [8]. Many other microRNAs have been found correlated with the prognosis of the GC patients. They are also considered as the proven biomarkers for GC with potential clinical value [9]. Along with the rapid development of high-throughput sequencing and the emergence of bioinformatics, our understanding about tumors has been greatly elevated. In the big data era, the mining of tumor information has deepened our knowledge of genomic changes associated with the complex diseases. An increasing number of potential biomarkers related to survival and prognosis have been developed through the mining of public databases. However, a single biomarker hardly achieves a good prediction effect, while a gene expression signature consisting of several genetic markers may improve the sensitivity and specificity of prediction. Prediction based on multiple genes can help the physicians to choose the best therapeutic regimen. However, many pathways are not being explored to identify novel biomarkers for GC. There exists a need to look for efficient and sensitive biomarkers for GC.

In the present study, TCGA database was utilized to uncover new prognostic biomarkers of GC patients [10]. Complete mRNA expression datasets were extracted from the GC patients in TCGA and Gene Expression Omnibus (GEO) databases [11]. A signature consisting of four genes that could accurately forecast the GC patients' prognosis was established in TCGA dataset and validated in the GEO dataset. To our delight, this glycolysis-related genic signature could effectively distinguish the GC patients showing favorable overall survival from those with poor prognosis.

## 2. Materials and Methods

### 2.1. Data Acquisition

The clinical information of 443 GC patients and mRNA expression data of 378 GC patients were downloaded from TCGA database (Table 1). The gene expression and clinical information data of 300 GC patients were retrieved from the GEO database (GSE62254). The clinical data include sex, age, survival time, overall survival status, grading, tumor-node-metastasis (TNM) staging, clinical T stage, clinical N stage, and clinical M stage.

### 2.2. Gene Set Enrichment Analysis (GSEA)

Gene Set Enrichment Analysis (GSEA) software was used to determine whether the identified gene set differs significantly between the GC group and the normal tissue group [12]. We used random seeds and the default parameters in the GSEA analysis. Next, the expression matrix by the composition of 321 mRNAs in GC samples and 33 paracancerous tissues samples used as controls downloaded from TCGA database were analyzed by the software, and five glycolysis-related gene sets were incorporated, respectively, for the analysis of the gene set enrichment significance. Finally, a normalized *P* value (*P* < 0.05) was considered statistically significant.

### 2.3. Survival Analysis

We used Kaplan-Meier survival analysis by survival package of R to evaluate the association between OS and genes' expression, age, TNM staging, T stage, N stage, and M stage. Log-rank method was used to determine the difference in overall survival between two subgroups of GC patients. For the univariate analysis, we used logistic regression model to analyze the association between OS and gene expression. We also used logistic regression model to perform multivariate analysis which determined the association between OS and gene expression as well as various clinical factors. The hazard ratio and the 95% confidence interval of hazard ratio were extracted from the logistic regression model.

### 2.4. The Construction of Risk Score

We used TCGA dataset to process training procedures and GEO dataset to process validation procedures. For the training procedure, first, we used median value of gene expression to divide the GC patients into high expression group and low expression group; candidate prognosis-associated genes were identified by univariate analysis.

The mRNAs were grouped into risk genes (hazard ratio, HR > 1) and protective genes (0 < HR < 1) [13]. Then, multivariate analysis was carried out to validate the association of the risk genes with overall survival after adjustment of clinical features. In order to obtain optimal gene combination to predict the prognosis, we used both-sided stepwise regression to analyze optimal risk gene combination by MASS package of R. As a result, the risk score formula was established by linear combination of the top four prognosis-associated genes with the lowest step Akaike Information Criterion (AIC) value of stepwise regression analysis, including *GMPPA*, *GPC3*, *NUP50*, and *VCAN*, using regression coefficients of multivariate Cox regression models.

(1) $$\begin{array}{c} \text{Risk score}=\overset{n}{\underset{i=1}{\sum }}{\text{Gene}}_{i}\times \text{Expression of }\beta_{i}. \end{array}$$

*β* was the coefficient derived from the multivariate regression models of TCGA cohort. For the validation procedure, we used the abovementioned model to perform multivariate analysis on the GEO dataset. Then, the prognostic significance of risk score was verified by Kaplan-Meier survival analysis and difference in survival curves was compared by log-rank method in TCGA and GEO datasets. The prognostic importance of risk score was further assessed by receiver operating characteristic (ROC) curve analysis in both TCGA and GEO datasets to evaluate the performance of the risk score model [14]. Then, the values of area under the curve (AUC) were determined accordingly for the risk score. *P* < 0.05 was considered statistically significant. All the analyses were performed in R.

### 2.5. Differential Expression and Mutation Analyses

To compare the expression level of certain risk genes between cases and controls of GC, the differential gene expression was determined by the Student *t*-test between the 33 adjacent noncancerous tissues and 238 GC tissues. Mutations and expression data of the selected genes were both obtained from TCGA dataset. All statistical analyses were conducted using R 3.6.2. *P* < 0.05 was considered statistically significant.

## 3. Results

### 3.1. GSEA-Based Glycolysis-Related Gene Sets Show Significant Differences between the Normal Gastric Samples and Tumor Samples

**Figure 1:**
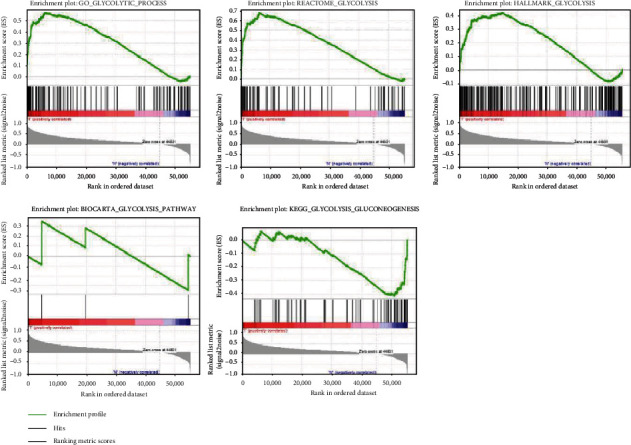
GSEA results of five gene set enrichment profiles (GO_GLYCOLYTIC_PROCESS, REACTOME_GLYCOLYSIS, HALLMARK_GLYCOLYSIS, BIOCARTA_GLYCOLYSIS_PATHWAY and KEGG_GLYCOLYSIS_GLUCONEOGENESIS).

The GSEA gene database was used to collect glycolysis-related gene sets (https://www.gsea-msigdb.org/gsea/msigdb/search.jsp). Five glycolysis-related gene sets were identified, including BIOCARTA_GLYCOLYSIS_PATHWAY, GO_GLYCOLYTIC_PROCESS, HALLMARK_GLYCOLYSIS, KEGG_GLYCOLYSIS_GLUCONEOGENESIS, and REACTOME_GLYCOLYSIS. In the next step, GSEA was used to analyze whether these five glycolysis-related gene sets were significantly differentially expressed between the GC tissues and adjacent noncancerous tissues. We found that GO_GLYCOLYTIC_PROCESS and REACTOME_GLYCOLYSIS were significantly differentially expressed between the precancerous tissues and GC tissues (*P* < 0.01). However, the differences in the other three glycolysis-related datasets were not statistically significant (*P* > 0.05, Table 2, Figure 1).

### 3.2. Identification of the Glycolysis-Related Genes Correlated to the Survival of GC Patients

We performed GSEA to screen the specific functional gene sets that were significantly differentially expressed in cancer tissues as compared to controls. Survival analysis was used to analyze which genes in these gene sets had an impact on the prognosis. Then, we performed differential gene expression analysis to verify whether prognostic genes are specifically expressed in cancer tissues.

In order to identify the novel biomarkers predicting the GC patients' prognosis, we first performed univariate Cox regression analysis on the glycolysis-related genes. Twenty-four genes were found significantly correlated to the OS of the GC patients (*P* < 0.05 for all cases). After the adjustment of clinical features, 18 independent genes were identified by multivariate analysis, including 10 protective genes and 8 risk genes. The GEO dataset validated that seven genes (GDP-mannose pyrophosphorylase A (*GMPPA*), glypican 3 (*GPC3*), NDC1 transmembrane nucleoporin (*NDC1*), nucleoporin 50 (*NUP50*), solute carrier family 35 member A3 (*SLC35A3*), tyrosylprotein sulfotransferase 1 (*TPST1*), and Versican (*VCAN*)) were significantly associated with overall survival in both cohorts (Table 3, Supplementary Table 1). The regression coefficients were calculated correspondingly.

We used both-sided stepwise regression to analyze optimal gene combination. The results showed that GMPPA, GPC3, NUP50, VCAN, and TPST1 and GMPPA, GPC3, NUP50, and VCAN both reached the best result with the lowest AIC value of 1488.9 among all combinations (Supplementary Figure 1A). Then, we calculated the risk score of the test set by two models, respectively, the AUC values of two models were 0.603 and 0.607 (Supplementary Figure 1B), which showed that the combination of GMPPA, GPC3, NUP50, and VCAN was better than the other combination. So, a prognostic prediction model based on the top four prognosis-associated glycolysis-related genes was established as follows:

(2) $$\begin{array}{c} \text{Risk score}=\left(0.49\times \text{Expression of GMPPA}\right)+\left(1.75\times \text{Expression of GPC}3\right)+\left(0.55\times \text{Expression of NUP}50\right)+\left(1.7\times \text{Expression of VCAN}\right). \end{array}$$

**Figure 2:**
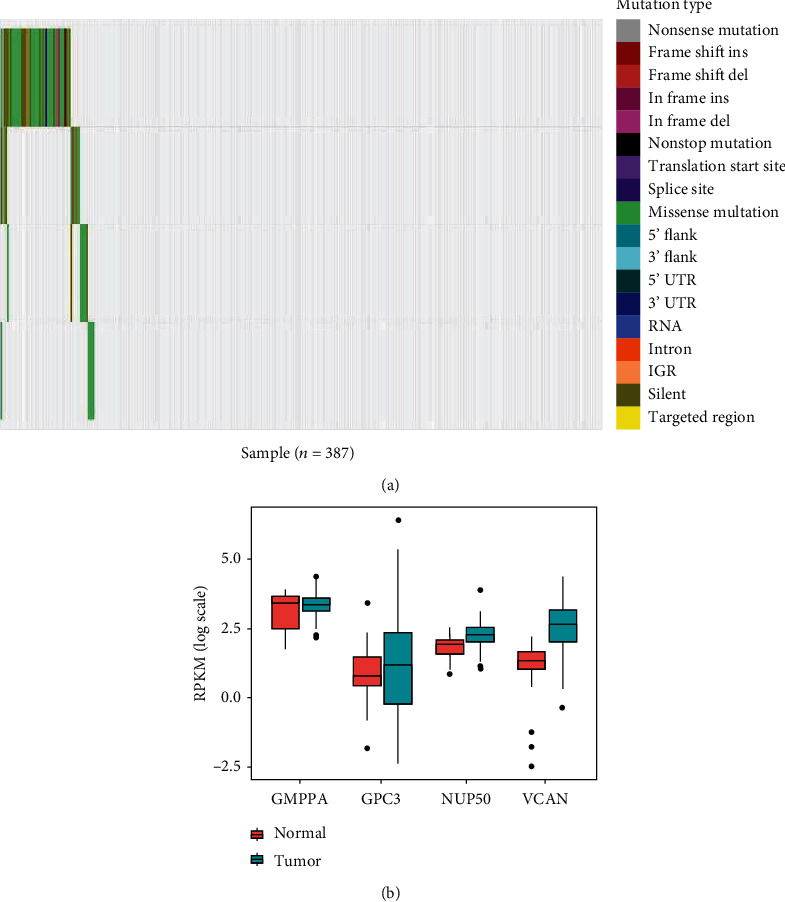
Identification of mRNAs correlated to survival of patients. (a) The mutation profile for the four genes in 387 GC samples. Ins: insertion; Del: deletion; UTR: untranslated region; IGR: intergenic region. (b) Expression difference of four genes between normal and GC tumor tissues.

Then, alterations in the expressions of these four genes of 378 GC patients were analyzed.  Figure 2(a) shows that the alterations in the four genes, *GMPPA*, *GPC3*, *NUP50*, and *VCAN*, were 2.58%, 1.3%, 1.8%, and 11.63%, respectively. The differential expression of these four genes in the GC tissues and normal tissues was further analyzed. The results showed that three genes (*GPC3*, *NUP50*, and *VCAN*) were highly expressed in the GC tissues, but lowly expressed in the normal tissues (*P* < 0.05 for all cases, Student's *t*-test, Figure 2(b)).

### 3.3. Relations between the Risk Score and Prognosis of GC Patients

**Figure 3:**
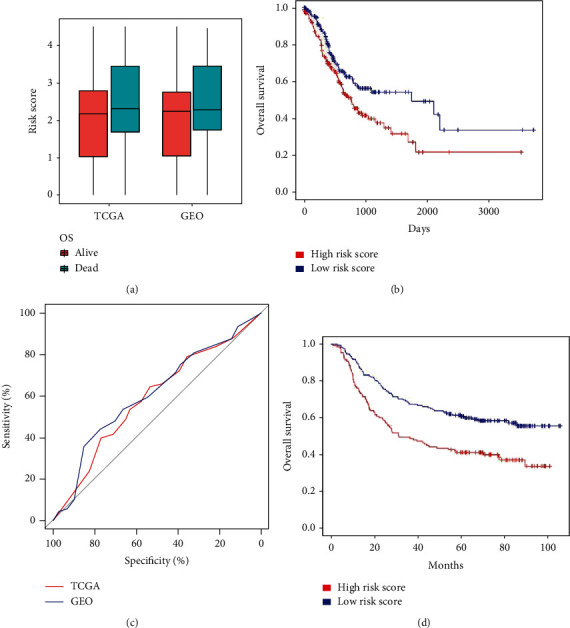
The four-gene signature predicts overall survival of the patients with GC. (a) Difference of mRNA risk score in deceased and alive GC patients. (b) Kaplan-Meier curve of patients in subgroups of GC patients with different overall survival risks in TCGA dataset. (c) ROC curves for the risk scores of TCGA and GEO datasets. (d) Kaplan-Meier curve of patients in subgroups of GC patients with different overall survival risks in the GEO dataset.

The GC patients were divided into the high-risk group and the low-risk group according to the median risk score.  Figure 3(a) shows the deceased GC patients showed significantly higher risk scores than alive GC patients (*P* < 0.05 for all cases, Student's *t*-test). Next, the value of these four mRNA signatures in GC prognosis was determined. The Kaplan-Meier curve showed that high risk scores were significantly associated with poor prognosis (*P* < 0.001, Figure 3(b)).  Figure 3(c) shows the AUC value of the risk score was 0.59 in TCGA dataset. Lastly, the negative correlation was validated between risk score and overall survival, with an AUC of 0.61 in the GEO cohort (Figures 3(c) and 3(d)).

### 3.4. The Risk Score Is an Independent Prognostic Indicator

In order to compare risk scores with conventional clinical features, univariate and multivariate analyses were conducted to estimate the significance of the above indicators in TCGA cohort. These indicators include risk score, age, gender, grading, and TNM staging. Our purpose was to compare the risk scores and the common clinical features. Univariate analysis revealed that age (HR: 1.02; 95% CI: 1.01~1.04; *P* < 0.01), TNM staging (HR: 1.58; 95% CI: 1.28~1.93; *P* < 0.01), clinical T stage (HR: 1.31; 95% CI: 1.07~1.61; *P* = 0.01), clinical N stage (HR: 1.34; 95% CI: 1.16~1.56; *P* < 0.01), clinical M stage (HR: 2.22; 95% CI: 1.28~3.86; *P* < 0.01), and risk score (HR: 1.22; 95% CI: 1.08~1.38; *P* < 0.01) were significantly correlated with the overall survival. However, gender and grading were uncorrelated to overall survival (*P* > 0.05 for all cases, Table 4). According to the multivariate analysis, risk score and age also significantly affected the prognosis (*P* < 0.05 for all cases, Table 4), indicating that these four genes were conducive to survival prediction. The univariate and multivariate analyses confirmed risk score was significantly negatively correlated with overall survival in the GEO cohort (*P* < 0.05 for all cases, Supplementary Table 2). Taken together, it is suggested that the risk score is a reliable prognostic predictor for GC.

### 3.5. The Four-Gene Risk Score Predicts Overall Survival Independently of Clinical Characteristics

**Figure 4:**
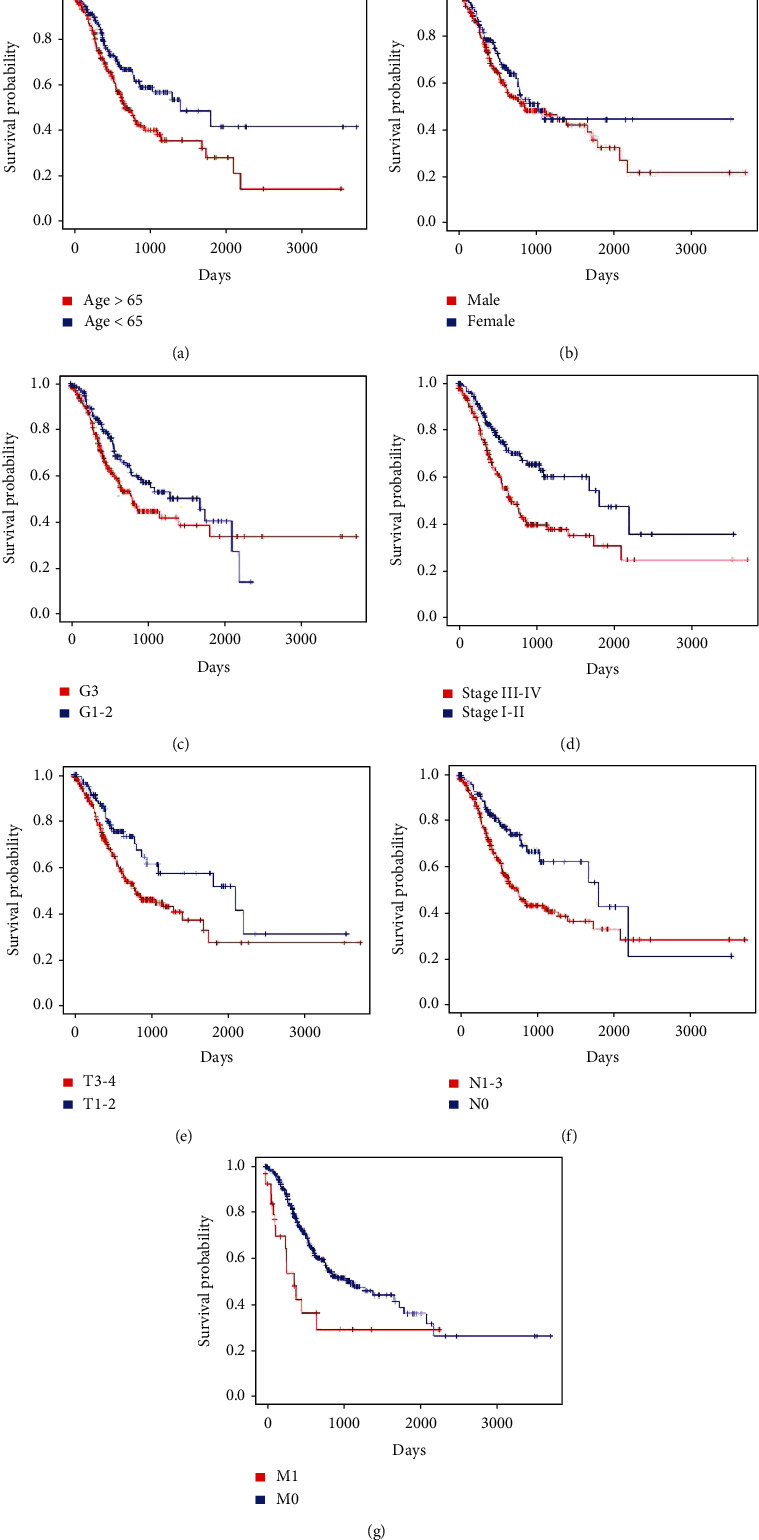
Kaplan-Meier survival analysis of clinical features and overall survival in GC patients in TCGA dataset ((a)–(g) represent age, gender, grade, TNM stage, T stage, N stage, and M stage).

Univariate analysis was performed to identify the influential factors of OS. Age, TNM staging, T stage, N stage, and M stage were significantly correlated with OS of GC patients in TCGA dataset (*P* < 0.05 for all cases, Figure 4). Neither gender nor grading was significantly correlated to the poor survival prognosis of the GC patients (*P* > 0.05 for all cases, Figure 4). As confirmed in the GEO cohort, TNM staging, T stage, N stage, and M stage were also significantly associated with OS of GC patients (*P* < 0.05 for all cases, Supplementary Figure 2).

**Figure 5:**
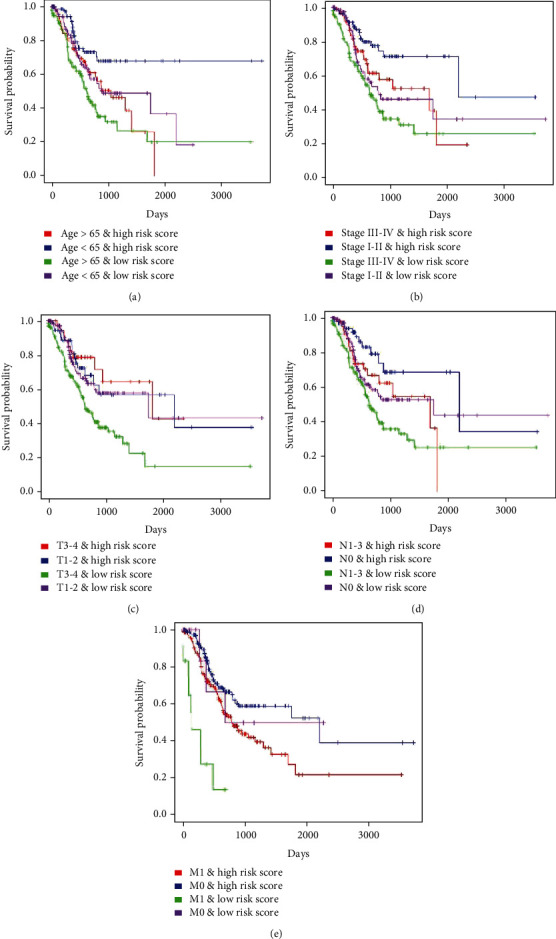
The Kaplan-Meier curves for the patient's risk score in subgroups of GS patients stratified by each clinical feature in TCGA cohort. GC patients were firstly divided into the high-risk score and low-risk score groups, each of which was further split into two subgroups stratified by each clinical feature. (a–e) Kaplan-Meier curves of four subgroups stratified by different combinations of age and risk score, TNM stage and risk score, T stage and risk score, N stage and risk sore, and M stage and risk score, respectively.

In order to verify the accuracy of our analysis, we used the Kaplan-Meier curve for stratification analysis of the above results. Results showed that the risk score was negatively associated with overall survival in the Stage I-II, T3-4, N1-3, M1, and M0 subgroups except for the Stage III-IV, T1-2, and N0 subgroups (*P* < 0.05 for all cases, Figure 5). The GEO cohort validated risk score was an independent negative prognostic factor in the Stage III-IV, T3-4, N0, N1-3, and M0 subgroups of GC patients except for the Stage I-II, T1-2, and M1 subgroups (*P* < 0.05 for all cases, Supplementary Figure 3). Thus, the risk score, to a large extent, might have a high value for survival prediction among GC patients independently of clinical characteristics.

## 4. Discussion

In the early 20^th^ century, German scientist Warburg discovered that when the cancer cells proliferate rapidly, glycolysis was the preferred metabolic pathway even there is an adequate supply of oxygen. This process provided energy and precursors needed for the synthesis of biomacromolecules in the cancer cells [15, 16]. Therefore, cancer cells have an intense uptake of glucose under aerobic conditions. The glycolysis-mediated energy production is known as the Warburg effect or aerobic glycolysis [17]. So far, a large number of studies have shown that the Warburg effect is closely related to tumor occurrence, development, and prognosis [18]. Previous studies have shown that the glycolysis in cancer cells is closely related to oncogene activation and cancer suppressor gene inactivation. But most of these studies have focused on the tumor occurrence, development mechanism, and pathogenesis [19–21]. In contrast, few researchers are devoted to the prognostic prediction of cancers based on the glycolysis-related genes. Moreover, most of these studies have focused on applying a single glycolysis-related biomarker to predict the prognosis of cancer patients, rather than a group of glycolysis-related genes.

With the rapid advance of gene sequencing technology, we are now able to extract gene expressions from the tumor samples to identify diagnostic and prognostic biomarkers for cancers. This is also the most common method at present [22]. Unlike the conventional approach, our study is aimed at looking for biomarkers with prognostic significance by data mining. First, we performed GSEA for the expressions of 321 mRNAs in 443 GC patients. We analyzed the differential expression of the glycolysis-related gene sets in the GC tissues and adjacent normal tissues. In order to identify genes with prognostic predictive value in GC patients, we performed univariate and multivariate Cox regression analyses. Based on our comprehensive analysis, the signature consisting of four glycolysis-related genes was identified. *GPC3* is a membrane-bound heparan sulfate proteoglycan and overexpressed in majority of hepatocellular carcinomas (HCC), 45% of squamous cell lung cancer cases, and 19% of head and neck squamous cell cancer cases [23]. It shows a relatively high diagnostic value for HCC [24]. In line with our study, elevated expression of *GPC3* is predictive of an inferior prognosis in HCC [25]. Anti-GPC3 antibody markedly inhibits the growth of HepG2 cells and promotes cellular apoptosis in HCC [26]. Additionally, *GPC3* is implicated in cellular protection against mitoxantrone in gastric carcinoma cell line PG85-257RNOV, characterized by reduced resistance to mitoxantrone and etoposide by anti-GPC3 ribozyme [27]. *VCAN* gene is related to epithelial-mesenchymal transition (EMT), which is a key step inducing distant metastasis of tumors. The high expression of *VCAN* is associated with the poor prognosis of leukemia patients. ShRNA-mediated silencing of *VCAN* can significantly inhibit the migration and invasion of the leukemia cells, which means that *VCAN* may be the novel diagnostic and therapeutic target for AML [28]. Mutations in three genes (*DNAJC2*, *GMPPA*, or *MMRN2*) are negatively associated with survival in lung adenocarcinoma [29]. In line with our study, Luo et al. identified 9 glycolysis-related genes (*BPNT1*, *DCN*, *FUT8*, *GMPPA*, *GPC3*, *LDHC*, *ME2*, *PLOD2*, and *UGP2*) and the risk score developed by the 9 genes was associated with a worse prognosis in gastric cancer [30]. *NUP50* is a nucleoplasmically oriented component of the nuclear pore complex with a role in protein export [31]. *NUP50* deletion was associated with abnormalities in p27(Kip1) expression and cell proliferation in the developing neuroepithelium in a mouse model [32]. These results in combination with our study support that *GMPPA*, *GPC3*, and *VCAN* are negative prognostic factors in various cancer types; these genes might provide novel therapeutic targets for cancer therapy.

As compared with the existing biomarkers for prognostic prediction, the signature was a combination of several genes, which showed some inherent benefits than a single gene. This gene expression signature displayed higher specificity for prognostic prediction and might serves as a tool for classification prediction of GC patients. As shown by the results of the Kaplan-Meier curve analysis, GC patients with a higher risk score were associated with a poor prognosis. These results implied that the risk score might be meaningful for prognostic prediction of GC patients in the long run. The risk score may provide a basis for the development of individualized therapies. Although the signature consisting of the four glycolysis-related genes was a reliable prognostic predictor for GC, our study had certain limitations. Firstly, during stratification analysis, certain clinical features, such as Stage III-IV, T1-2, might affect the predictive capability of risk score for GC patients. One possible reason is that the sample size within subgroups is relatively small, which led to the unreliable prediction. Also, we knew little about certain genes as to their regulatory roles in glycolysis and the influence on prognosis. Moreover, these genes are not only involved in the glycolysis process, but the function of four risk genes may also affect the prognosis by the effort of cell adhesion and extracellular protein group expressions. Therefore, further study is needed, which also provides a new method for us to study the Warburg effect of GC.

In summary, a gene expression signature consisting of four glycolysis-related genes was constructed using the bioinformatics technology, and these genes were associated with the OS of GC patients. We verified that this gene expression signature was able to predict the prognosis of GC patients. Patients with a higher risk score were associated with worse prognosis. Our findings revealed the regulatory mechanism of specific genes in the glycolysis and its effect on the prognosis of GC.

## Figures and Tables

**Table 1:** Clinical data of GC patients (*n* = 443) obtained from The Cancer Genome Atlas.

Variables	Patients, *n* (%)
Sex	443
Male	285 (64.33%)
Female	158 (35.67%)
Age (years)	
≤65	197 (44.47%)
>65	241 (54.4%)
Grade	
G1	12 (2.7%)
G2	159 (35.89%)
G3	263 (59.36%)
Gx	9 (2.03%)
TNM stage	
I	59 (13.31%)
II	130 (29.34%)
III	183 (41.30%)
IV	44 (9.93%)
Unknown	28 (6.32%)
T stage	
T1	23 (5.19%)
T2	93 (20.99%)
T3	198 (44.69%)
T4	119 (26.86%)
TX	10 (2.25%)
N stage	
N0	132 (29.79%)
N1	119 (26.86%)
N2	85 (19.18%)
N3	88 (19.86%)
NX	17 (3.83%)
Unknown	2 (0.45%)
M stage	
M0	391 (88.26%)
M1	30 (6.77%)
MX	22 (4.49%)

TX, NX, and MX are unknown cancer stages.

**Table 2:** Gene sets enriched in GC (412 samples).

GS follow link to MSigDB	Size	NES	NOM	FDR
			*P* value	*q* value
GO_GLYCOLYTIC_PROCESS	106	1.91	0.006	0.006
REACTOME_GLYCOLYSIS	72	1.97	0.004	0.004
HALLMARK_GLYCOLYSIS	200	1.36	0.144	0.144
BIOCARTA_GLYCOLYSIS_PATHWAY	3	0.58	0.941	0.941
KEGG_GLYCOLYSIS_GLUCONEOGENESIS	62	-1.30	0.182	0.182

**Table 3:** Four prognostic genes were selected via univariable and multivariable Cox regression analysis.

Gene	Univariate analysis			Multivariate analysis		
	HR	95% CI	*P* value	HR	95% CI	*P* value
*GMPPA*	0.63	0.45-0.88	<0.01	0.49	0.34-0.7	<0.01
*GPC3*	1.8	1.29-2.51	<0.01	1.75	1.21-2.55	<0.01
*NUP50*	0.66	0.47-0.91	0.01	0.55	0.38-0.8	<0.01
*VCAN*	1.67	1.2-2.33	<0.01	1.7	1.18-2.47	<0.01

**Table 4:** Univariable and multivariable analyses for each clinical feature.

Clinical feature	Univariate analysis			Multivariate analysis		
	HR	95% CI	*P* value	HR	95% CI	*P* value
Age	1.02	1.01-1.04	<0.01	1.03	1.01-1.05	<0.01
Gender	1.24	0.87-1.75	0.23	1.34	0.91-1.97	0.13
Grade	1.37	1.00-1.89	0.05	1.30	0.90-1.88	0.16
Stage	1.58	1.28-1.93	<0.01	1.38	0.92-2.08	0.12
T	1.31	1.07-1.61	0.01	0.98	0.73-1.32	0.89
M	2.22	1.28-3.86	<0.01	1.68	0.77-3.67	0.19
N	1.34	1.16-1.56	<0.01	1.11	0.8-1.40	0.36
Risk score	1.22	1.08-1.38	<0.01	1.51	1.04-2.21	0.03

## Data Availability

All data comes from TCGA database (https://portal.gdc.cancer.gov/) and the GEO database (https://www.ncbi.nlm.nih.gov/geo/), and all the data are reliable.

## Abbreviations

GC: Gastric cancer
TCGA: The Cancer Genome Atlas
GEO: Gene Expression Omnibus
GSEA: Gene Set Enrichment Analysis
OS: Overall survival
HR: Hazard ratio.

## Supplementary Materials

Supplementary 1 Supplementary Figure 1: (A) AIC value of stepwise regression at each step. (B) ROC curves for the risk scores of *GMPPA*, *GPC3*, *NUP50*, *VCAN*, and *TPST1* and *GMPPA*, *GPC3*, *NUP50*, and *VCAN* in the GEO datasets.

Supplementary 2 Supplementary Figure 2: Kaplan-Meier survival analysis for patients with GC in the GEO dataset (A–F represent age, gender, TNM stage, T stage, N stage, and M stage, respectively).

Supplementary 3 Supplementary Figure 3: Kaplan-Meier curves for the patient's risk score in subgroups of GS patients stratified by each clinical feature in the GEO cohort (A–D denote TNM stage, T stage, N stage, and M stage, respectively).

Supplementary 4 Supplementary Table 1: seven prognostic genes were selected via univariable and multivariable Cox regression analysis in the GEO dataset. Supplementary Table 2: univariable and multivariable analyses for each clinical feature in the GEO dataset.
