# Supplementary figures and images for: Identifying Novel Cell Glycolysis-Related Gene Signature Predictive of Overall Survival in Gastric Cancer
Source: Biomed Res Int. 2021 Mar 12;2021:9656947. doi: 10.1155/2021/9656947 (PMC7982000; doi:10.1155/2021/9656947)

**A**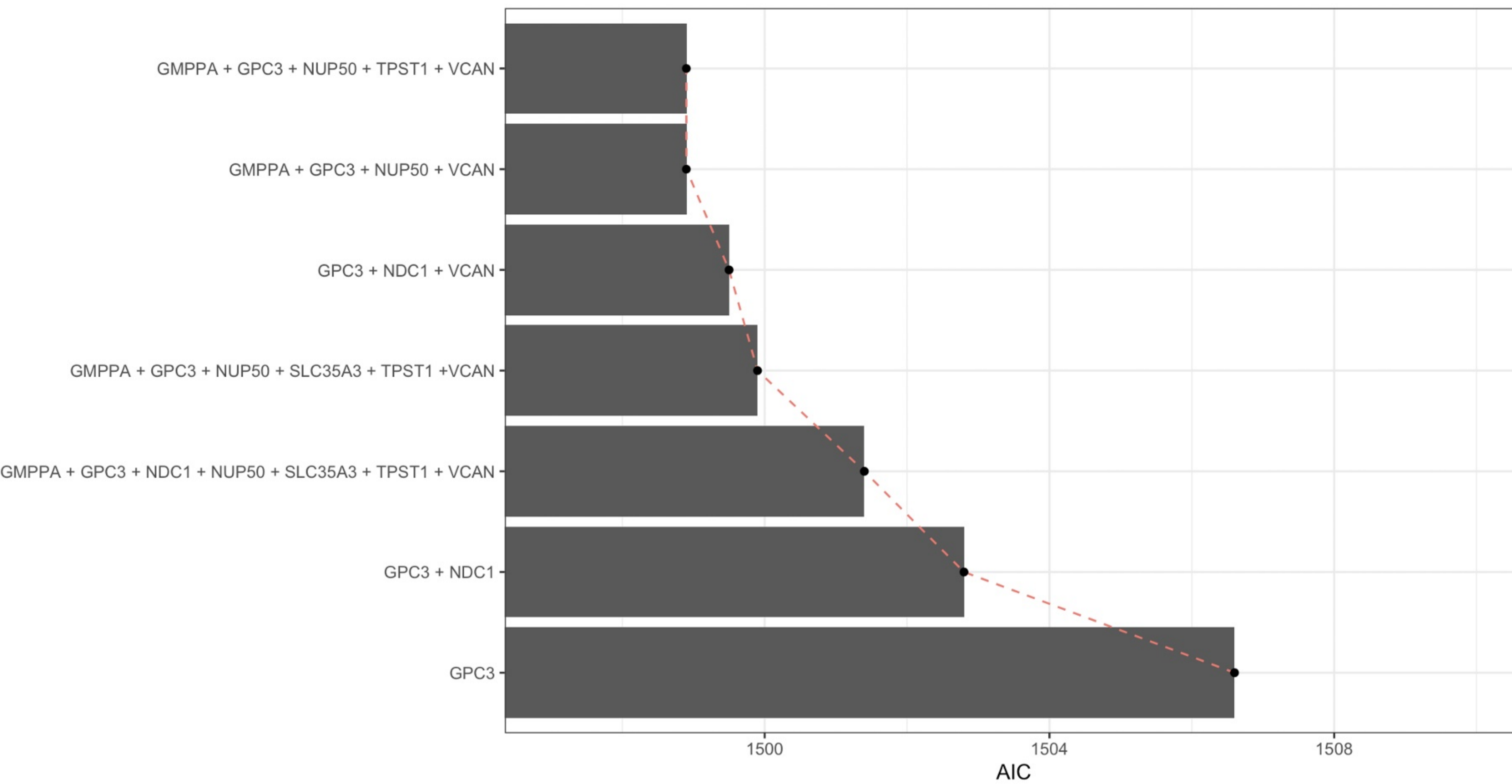**B**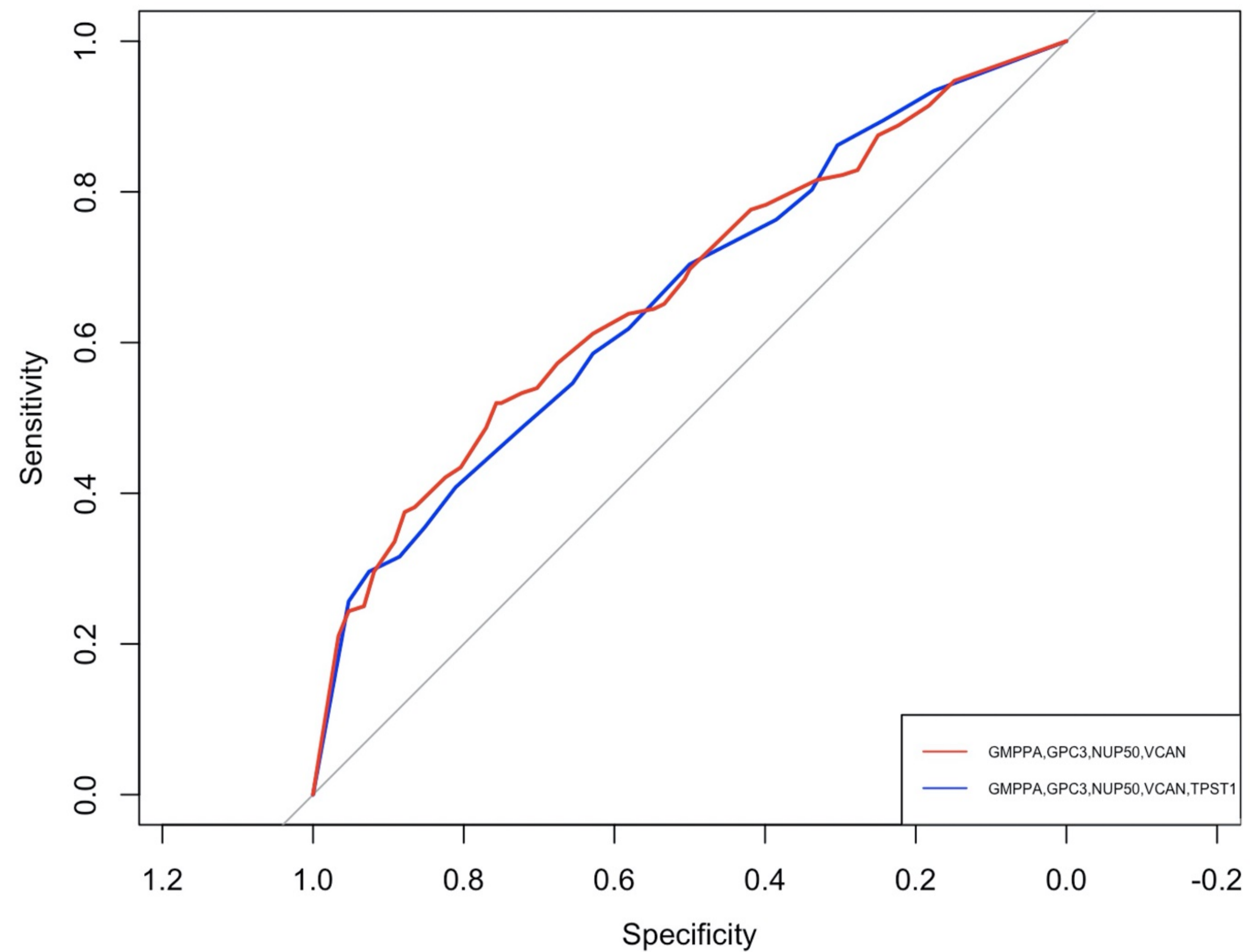

Supplement: Supplementary 1 — Supplementary Figure 1: (A) AIC value of stepwise regression at each step. (B) ROC curves for the risk scores of GMPPA, GPC3, NUP50, VCAN, and TPST1 and GMPPA, GPC3, NUP50, and VCAN in the GEO datasets. [file 9656947.f1.pdf]

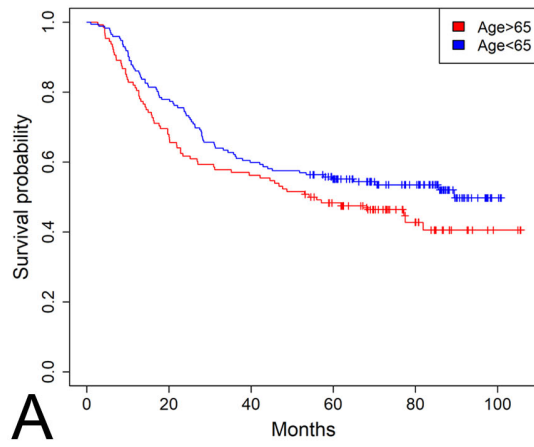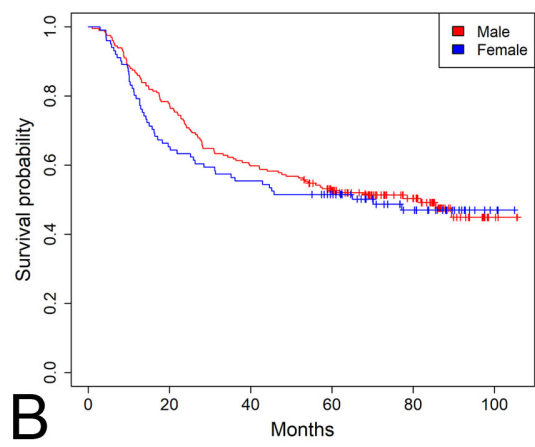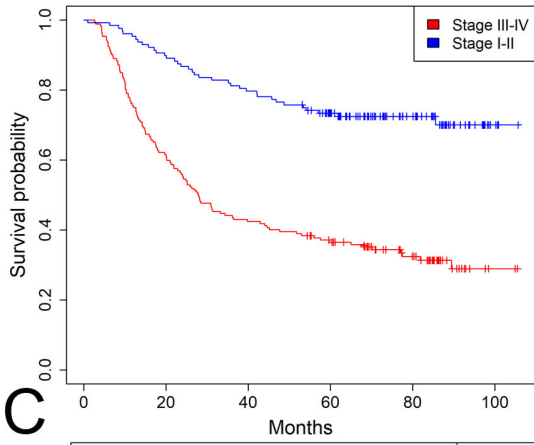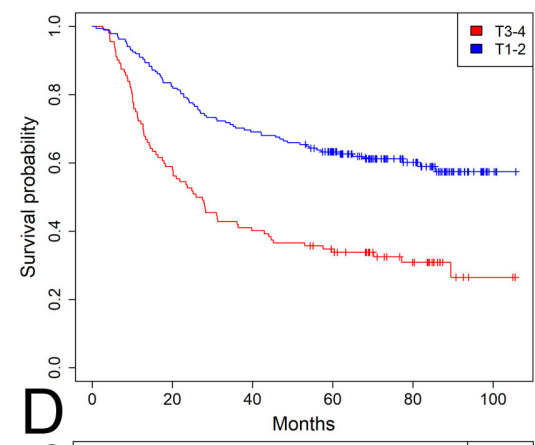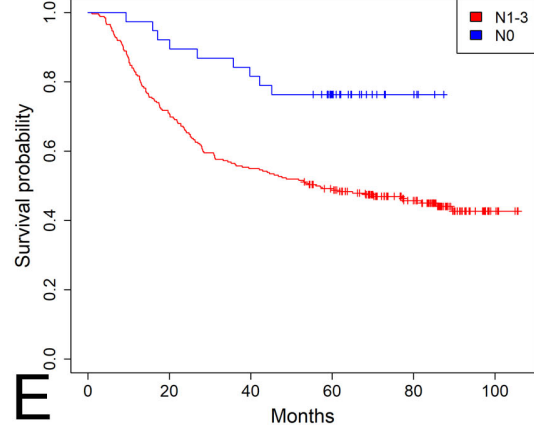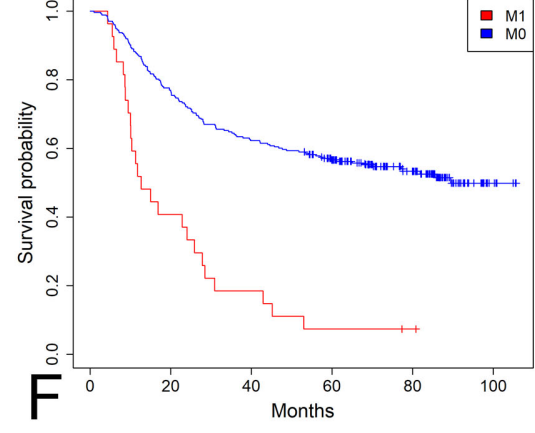

Supplement: Supplementary 2 — Supplementary Figure 2: Kaplan-Meier survival analysis for patients with GC in the GEO dataset (A–F represent age, gender, TNM stage, T stage, N stage, and M stage, respectively). [file 9656947.f2.pdf]

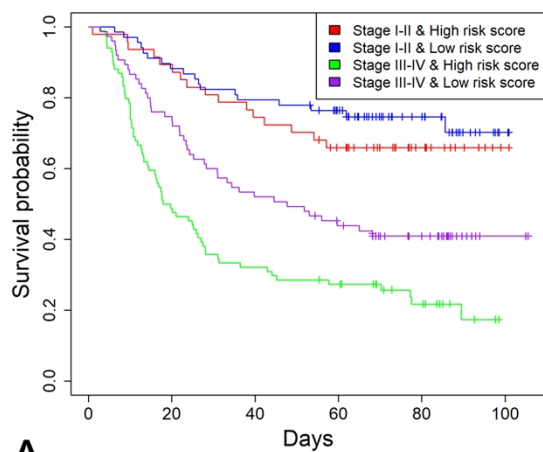

A

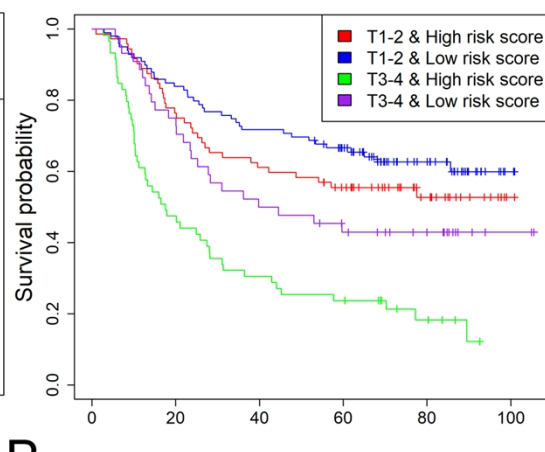

B

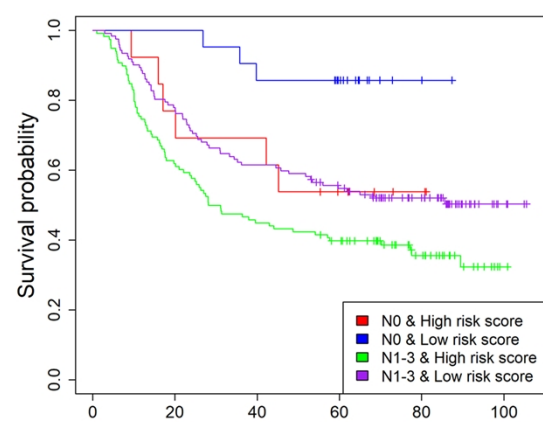

C

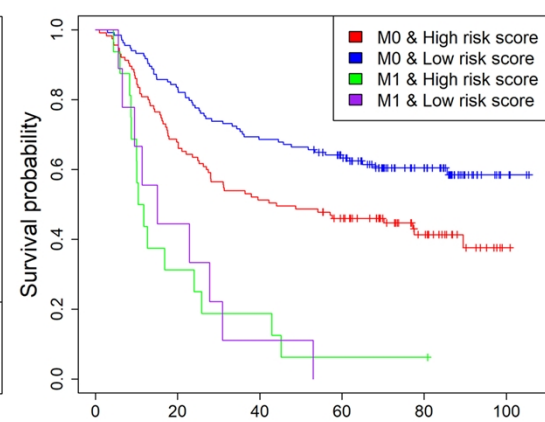

D

Supplement: Supplementary 3 — Supplementary Figure 3: Kaplan-Meier curves for the patient's risk score in subgroups of GS patients stratified by each clinical feature in the GEO cohort (A–D denote TNM stage, T stage, N stage, and M stage, respectively). [file 9656947.f3.pdf]
