# Supplementary material for: Identifying Novel Cell Glycolysis-Related Gene Signature Predictive of Overall Survival in Gastric Cancer
Source: Biomed Res Int. 2021 Mar 12;2021:9656947. doi: 10.1155/2021/9656947 (PMC7982000; doi:10.1155/2021/9656947)
Supplement: Supplementary 4 — Supplementary Table 1: seven prognostic genes were selected via univariable and multivariable Cox regression analysis in the GEO dataset. Supplementary Table 2: univariable and multivariable analyses for each clinical feature in the GEO dataset. [file 9656947.f4.docx]

Supplementary Table1. Seven prognostic genes were selected via univariable and multivariable Cox regression analysis in the GEO dataset

|  | Univariate analysis | | | Multivariate analysis | | |
| --- | --- | --- | --- | --- | --- | --- |
| Gene | HR | 95% CI | P value | HR | 95% CI | P value |
| GMPPA | 0.62 | 0.45-0.86 | <0.01 | 0.66 | 0.47-0.91 | <0.01 |
| GPC3 | 2 | 1.45-2.78 | <0.01 | 1.73 | 1.24-2.41 | <0.01 |
| *NUP50* | 0.62 | 0.45-0.85 | <0.01 | 0.7 | 0.5-0.98 | 0.04 |
| *VCAN* | 1.4 | 1.02-1.93 | 0.04 | 1.46 | 1.05-2.02 | 0.02 |
| *NDC1* | 0.63 | 0.45-0.87 | <0.01 | 0.63 | 0.44-0.91 | 0.01 |
| *SLC35A3* | 0.66 | 0.48-0.92 | 0.01 | 0.60 | 0.41-0.87 | <0.01 |
| *TPST1* | 1.62 | 1.17-2.26 | <0.01 | 1.52 | 1.06-2.19 | 0.02 |

Supplementary Table2. Univariable and multivariable analyses for each clinical feature in the GEO dataset

|  | Univariate analysis | | | Multivariate analysis | | | | |
| --- | --- | --- | --- | --- | --- | --- | --- | --- |
| Clinical feature | HR | 95% CI | P value | HR | 95% CI | P value | | |
| Gender | 1.17 | 0.82-1.67 | 0.38 | 0.90 | 0.65-1.27 | | 0.56 |  |
| Age | 1.03 | 1.01-1.04 | <0.01 | 1.01 | 1.00-1.03 | | 0.18 |  |
| T | 1.24 | 0.93-1.66 | 0.14 | 1.79 | 1.44-2.22 | | <0.01 |  |
| N | 1.47 | 1.07-2.01 | 0.02 | 1.96 | 1.63-2.34 | | <0.01 |  |
| M | 2.01 | 1.20-3.38 | 0.01 | 3.84 | 2.48-5.94 | | <0.01 |  |
| Stage | 1.45 | 0.97-2.16 | 0.07 | 2.23 | 1.84-2.7 | | <0.01 |  |
| Risk_score | 1.77 | 1.28-2.46 | <0.01 | 1.28 | 1.11-1.47 | | <0.01 |  |
